# Association of Weight-Adjusted Body Fat and Fat Distribution with Bone Mineral Density in Middle-Aged Chinese Adults: A Cross-Sectional Study

**DOI:** 10.1371/journal.pone.0063339

**Published:** 2013-05-20

**Authors:** Yan-hua Liu, Ying Xu, Ya-bin Wen, Ke Guan, Wen-hua Ling, Li-ping He, Yi-xiang Su, Yu-ming Chen

**Affiliations:** Guangdong Provincial Key Laboratory of Food, Nutrition and Health, School of Public Health, Sun Yat-sen University, Guangzhou, People’s Republic of China; University of Southampton, United Kingdom

## Abstract

**Background:**

Although it is well established that a higher body weight is protective against osteoporosis, the effects of body fat and fat distribution on bone mineral density (BMD) after adjustment for body weight remains uncertain.

**Objective:**

To examine the relationship between body fat and fat distribution and BMD beyond its weight-bearing effect in middle-aged Chinese adults.

**Method:**

The study had a community-based cross-sectional design and involved 1,767 women and 698 men aged 50–75 years. The BMD of the lumbar spine, total hip, and whole body, and the fat mass (FM) and percentage fat mass (%FM) of the total body and segments of the body were measured by dual-energy X-ray absorptiometry. General information on the participants was collected using structured questionnaire interviews.

**Result:**

After adjusting for potential confounders, an analysis of covariance showed the weight-adjusted (WA-) total FM (or %FM) to be negatively associated with BMD in all of the studied sites (*P*<0.05) in both women and men. The unfavorable effects of WA-total FM were generally more substantial in men than in women, and the whole body was the most sensitive site related to FM, followed by the total hip and the lumbar spine, in both genders. The mean BMD of the lumbar spine, total hip, and whole body was 3.93%, 3.01%, and 3.65% (in women) and 5.02%, 5.57%, 6.03% (in men) lower in the highest quartile (vs. lowest quartile) according to the WA-total FM (all p<0.05). Similar results were noted among the groups for WA-total FM%. In women, abdominal fat had the most unfavorable association with BMD, whereas in men it was limb fat.

**Conclusion:**

FM (or %FM) is inversely associated with BMD beyond its weight-bearing effect. Abdominal fat in women and limb fat in men seems to have the greatest effect on BMD.

## Introduction

Osteoporosis is an important public health problem in an aging society, because it has a high prevalence [1] and is associated with bone fractures [2] that can result in death or permanent disability [3]. Ample epidemiological data have shown that body weight or BMI is the predominant determinant of bone mineral density (BMD) or bone mineral content (BMC) [4], [5], and that a reduction in body weight can lead to bone loss [6]. A straightforward explanation for this finding is that a greater body weight imposes greater mechanical stress on bone, and bone mass increases in response to accommodate the greater load [7]. Many epidemiologic studies have found that fat mass (FM), which is a major component of body weight, is positively related to BMD (or BMC) [8]–[10]. Because adipose tissue is not simply an inert organ for energy storage but also expresses and secretes a variety of biologically active molecules, its effect on the skeleton may be influenced not only by weight bearing but also by other non-weight-bearing effects [11]. It is uncertain whether FM has a benefit on bone mass after the exclusion of its weight-bearing effect.

Few studies have examined the net effect of FM on bone mass after adjustment for weight. In a cross-sectional study of 7,137 Chinese men, 4,585 premenopausal women, and 2,248 postmenopausal women across a 5-kg strata of body weight, FM was significantly and inversely associated with the BMC of the whole body and total hip [12]. Another study found that the weight-adjusted abdominal FM was inversely associated with BMD in older Puerto Rican adults after confounders and the mechanical loading effects of weight were controlled for [13]. The results of these studies suggest that FM may have an unfavorable effect on bone mass. However, the studies did not determine whether the FM-associated lower bone mass was due to a greater FM or a lower lean mass (LM).

It is well recognized that there are gender differences in body fat distribution, with men tending to accumulate more visceral tissue and women tending to have more peripheral or subcutaneous adipose tissue [14]. Several studies have found that visceral fat has a more significant effect than subcutaneous fat on metabolic syndrome, cardiovascular risk factors, insulin resistance, dyslipidemia, and hypertension [15]–[17]. However, it is unclear whether abdominal fat is more effective than limb fat, and whether abdominal fat has a similar effect on bone mass in men and women after adjustment for body weight.

This community-based study aimed to determine the association of body fat and fat distribution with the BMD of the lumbar spine, hip, and whole body after adjusting for mechanical loading effects and other covariates in middle-aged Chinese men and women.

## Participants and Method

### Participant Recruitment

The community-based cross-sectional study was based on a cohort study designed to assess the determinants of cardiometabolic outcomes and osteoporosis, to which 3,169 participants aged between 50 and 75 years were recruited between July 2008 and June 2010. The participants were required to have been residents of Guangzhou (a large city in South China) for at least five years. Participants were recruited by sending invitation letters to residential buildings, posting local advertisements, giving health talks, and from referrals in the local community. Participants who reported having confirmed diabetes, cardiovascular disease, renal failure, cancer, metabolic bone disease, chronic glucocorticoid use, or a history of spine or hip fracture were excluded. In the follow-up survey, 704 participants dropped out due to refusal (433 persons), lost-to contact or emigration (194 persons), incompletion of the assessments of BMD or FM (41 persons) and serious diseases or death (36 cases). A total of 2,465 (77.8%) participants completed the first follow-up survey and BMD and body fat examinations between April 2011 and January 2013 at a mean (SD) interval of 3.2 (0.5) years of follow up. Cross-sectional data of the follow-up study were used for this study. The study protocol was approved by the Ethics Committee of the School of Public Health at Sun Yat-sen University. Written informed consent was obtained from all of the participants at initial enrollment and at the follow-up.

### Data Collection

Eligible subjects were invited to the School of Public Health of Sun Yat-sen University. Face-to-face interviews by trained interviewers were conducted to confirm their eligibility and to collect information on their socio-demographic characteristics, lifestyle, and diet using structured questionnaires. Anthropometric measurements were taken at baseline and follow-up. Dual-energy X-ray absorptiometry (DXA) measurements were performed at follow-up.

### Outcome Assessment

DXA (Discovery W; Hologic Inc., Waltham, MA, USA) was used to measure the BMD (g/cm^2^) of the lumbar spine (L_1_–L_4_), left total hip, and whole body. The scans for the lumbar spine and left hip were performed using a high-definition mode, whereas the scans for the whole body were performed in the default mode. The scans were analyzed with Hologic Discovery software version 3.2, and were performed by the same well trained professionals. The in-vivo coefficients of variation of the duplicated BMD measurements in 30 participants after re-positioning were 0.87%, 1.02%, and 1.18% for the lumbar spine, total hip, and whole body, respectively. The long-term coefficient of variation of the measurements was 0.29% as calculated by testing the phantom daily between April 2011 and January 2013.

### Exposure Assessment

**Figure 1:**
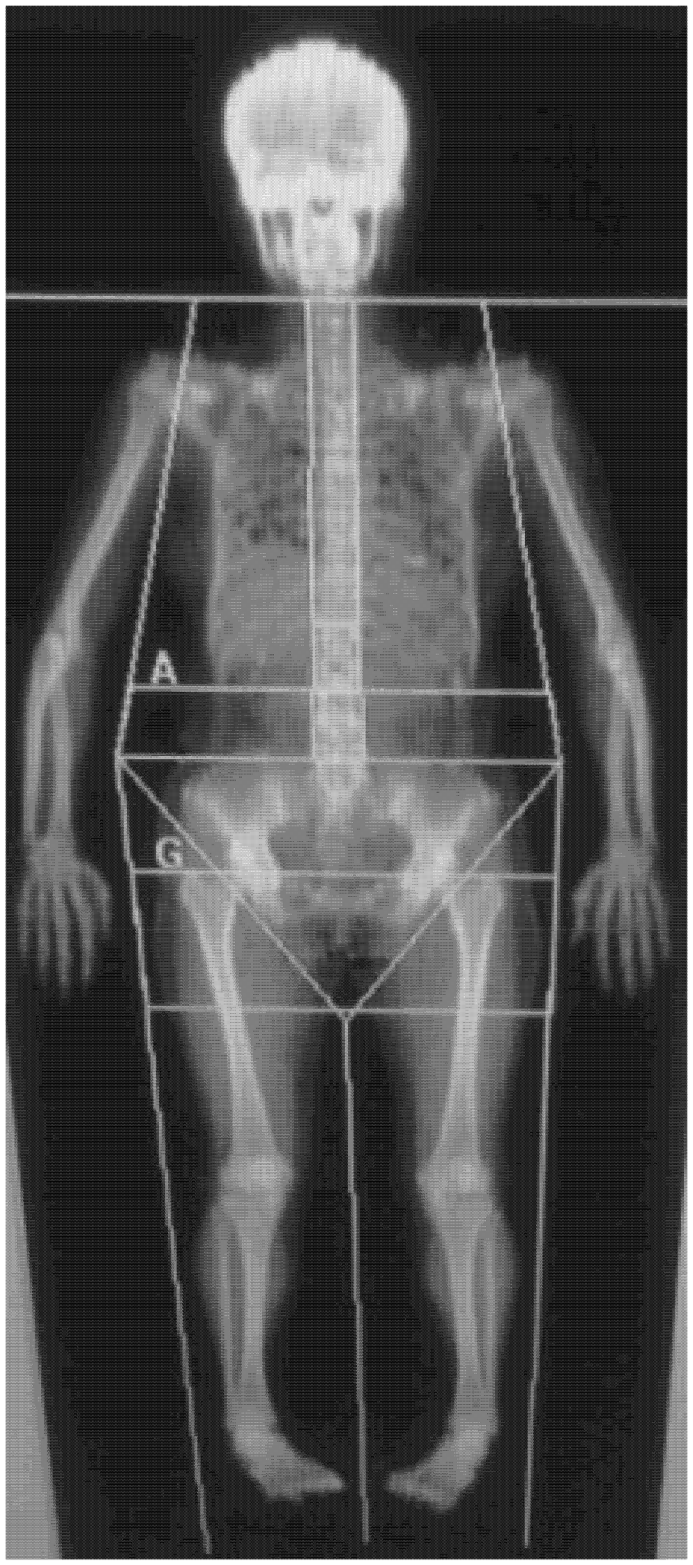
Regions of trunk fat, android (A) fat, and arm and leg fat as assessed by DXA. The trunk region is defined as the area between an upper horizontal border below the chin and a lower border formed by oblique lines passing through the hip joints. The android (A) region is defined as the area between a lower boundary at the pelvis cut and an upper boundary above the pelvis that is 20% of the distance between the pelvis and the neck cuts.

The height and weight of the participants were measured to the nearest 0.1 cm and 0.1 kg, respectively, without shoes and in light clothing. The FM and %FM of the whole body, trunk region, android region, and limbs and the total LM were obtained from the whole body DXA scans. The trunk region was defined as the area between an upper horizontal border below the chin and a lower border formed by oblique lines passing through the hip joints. The android region was defined as the area between a lower boundary at the pelvis cut and an upper boundary above the pelvis cut that is 20% of the distance between the pelvis and the neck cuts. The FM and %FM of the limbs were calculated from the fat mass and total mass of the bilateral arms and legs (FIG. 1). The root mean square precision of these measurements was 1.31% for the total FM and 1.20% for the total %FM.

### Covariates Assessment

Information on the demographics, education, alcohol consumption, cigarette smoking habits, physical activity (including average hours spent sitting, standing, walking, engaging in mild and vigorous physical activity, carrying a load, and walking upstairs), the history of fractures and other important diseases, medication, age at menarche (for women only) and years since menopause (YSM, where menopause is defined as the natural cessation of menstrual periods for more than 12 months, for women only) was collected from the structured questionnaires [18]. Participants who smoked at least one cigarette per day or drank alcohol once a week for at least six months were defined as smokers or drinkers. The assessment of the dietary intake of energy, protein, calcium (Ca), and phosphorus (P) was assessed based on a quantitative food frequency questionnaire (FFQ) that included 79 food groups or items that has been validated and described in previous reports [19]. The mean food intake per day, week, month, or year was reported at the interviews using the year before the interview as the reference period. Food pictures and full-scale food portions were provided as visual aids for the participants to judge portion size. Nutrients were calculated from the 2002 China Food Composition Table [20].

### Statistical Analyses

All of the analyses were performed separately for the male and female participants. The data were presented as means and SDs for the continuous variables and as frequencies for the categorical variables. A student’s *t*-test was used to ascertain the significance of the differences in the continuous variables between men and women, and Pearson’s Chi-square test was used to determine the significance of the differences in the categorical variables between the two genders. Because of the significant colinearity of body weight and FM (or %FM), we first regressed each FM index on body weight and saved the residuals. These residuals represent the variation in the FM indices that is independent of body weight. The residuals were added to the predicted value of the FM at the mean body weight to arrive at the weight-adjusted (WA-) indices of FM [21]. The study participants were divided into quartiles [Q1 (lowest), Q2, Q3, and Q4] according to their WA-indices of FM. The middle two quartiles (Q2 and Q3) were then re-combined into one category (Q2–Q3).

Univariate and multivariate analyses of variance (ANOVA and ANCOVA) were used to compare the mean BMDs of the lumbar spine, total hip, and whole body among the quartiles according to the WA-indices of FM. In the ANCOVA models, adjustments were made for age, education, energy-adjusted (EA-) intake of protein, calcium, and phosphorus, calcium supplements, daily energy expenditure in various physical activities except for sitting and lying, drinking and smoking (for men only), and estrogen use, age at menarche, and years since menopause (YSM) (for women only). To adjust for the confounding influence of the mechanical loading of body weight and skeletal size, we additionally adjusted for weight (except in the weight analysis) and height according to the method reported by Willett, et al [21]. Pair-wise comparisons were performed using the Bonferroni test. The WA-indices of FM were converted to a standard normal Z-score by gender, and then simple and multiple linear regression models were used to test for a linear trend in the relationship between the WA-indices of FM and BMD. The same covariates were adjusted for in the multiple linear regression models. The analyses were performed with SPSS 13.0 for Windows (SPSS, Inc., Chicago, USA). The formal hypothesis testing was two-sided, and the nominal type I error rate was 0.05.

## Results

A total of 2,465 participants (1,767 women and 698 men) were included in the analysis. The characteristics of the participants are shown in Table 1. The mean age of the participants was 59.6±4.7 years for the women and 62.2±5.3 years for the men. The BMD at all of the studied sites was higher in men than in women (*P*<0.001). However, the FM and FM% of the whole body, android region, trunk region, and limbs tended to be higher in the women than the men (*P*<0.001, except for the android region FM, *P* = 0.076). The women had a higher ratio of FM to LM than men, but a lower trunk to limb ratio of %FM.

**Table 1:** Characteristics of the study participants.

Variable	Female			Male			
	Mean	SD	N	Mean	SD	N	*P*
Age (y)	59.62	4.72	1767	62.17	5.27	698	<0.001
Weight (kg)	56.38	8.54	1767	66.30	9.76	698	<0.001
Fat indices							
Total fat (kg)	20.12	4.98	1767	16.66	4.89	698	<0.001
Total fat% (%)	35.59	4.47	1767	25.07	4.35	698	<0.001
Android fat (kg)	1.62	0.51	1767	1.57	0.64	698	0.076
Android fat% (%)	37.35	5.85	1767	29.86	6.45	698	<0.001
Trunk fat (kg)	10.06	2.84	1767	9.08	3.07	698	<0.001
Trunk fat% (%)	36.16	5.31	1767	27.38	5.41	698	<0.001
Limb fat (kg)	9.09	2.40	1767	6.49	1.91	698	<0.001
Limb fat% (%)	37.10	5.20	1767	22.74	4.21	698	<0.001
Trunk to limb%FM ratio	0.98	0.13	1767	1.21	0.14	698	<0.001
FM to LM ratio	0.56	0.11	1767	0.34	0.08	698	<0.001
Bone mineral density							
Lumbar spine(g/cm^2^)	0.850	0.145	1767	0.962	0.148	698	<0.001
Total hip(g/cm^2^)	0.804	0.112	1767	0.909	0.112	698	<0.001
Whole body(g/cm^2^)	1.062	0.103	1767	1.180	0.101	698	<0.001
Age at menarche (y)	13.99	1.68	1767	–	–	–	–
YSM (y)	9.98	6.30	1723	–	–	–	–
Estrogen (%, n, N)	6.3	109	1722	–	–	–	–
Education							<0.001
<6 years(%, n, N)	6.60	116	1749	5.30	36	684	
6–12 years(%, n, N)	72.30	1264	1749	62.10	425	684	
>12 years(%, n, N)	21.10	369	1749	32.60	223	684	
Smoking(%, n, N)	0.60	10	1759	28.30	197	696	<0.001
Drinking(%, n, N)	3.90	68	1759	15.70	109	696	<0.001
Calcium tablet(%, n, N)	33.90	596	1758	20.80	145	696	<0.001
Dietary intake							
Energy expenditure(kcal/d)	872.99	315.17	1759	1317.66	591.28	695	<0.001
Energy intake(kcal/d)	1666.51	463.54	1759	1983.61	557.75	696	<0.001
Protein (g)	68.41	21.66	1759	77.84	26.16	696	<0.001
Calcium (Ca, mg)	626.14	250.32	1759	612.27	255.83	696	0.219
Phosphorus (P, mg)	1069.30	321.61	1759	1206.68	371.76	696	<0.001

FM: fat mass; LM: lean mass; BMD: bone mineral density; YSM: years since menopause;

Energy expenditure: daily energy expenditure excluded sitting and lying.

In the univariate analyses, we generally found a dose-dependent positive association between most adiposity indices and BMD in both men and women, except a significantly inverse association between limb %FM and BMD at the whole body and spine, and between total %FM and trunk %FM and whole body BMD (data not shown). The multivariate analyses showed weight to be positively and linearly associated with the BMD of the lumbar spine, total hip, and whole body both in the men and the women (*P*<0.001, Table 2–4). The WA-total FM and WA-total FM% were significantly and inversely correlated with BMD at all of the studied sites in the men and the women after adjusting for mechanical loading effects and other potential confounders (*P*<0.05). In general, the unfavorable effects of WA-FM or WA-FM% on BMD were more substantial in the men than in the women. Whole body was the most sensitive site, followed by total hip and lumbar spine, in both genders. The mean BMD of the lumbar spine, total hip, and whole body was 3.93%, 3.01%, and 3.65% (in the women) and 5.02%, 5.57%, 6.03% (in the men) lower in the highest quartile (vs. lowest quartile) group by WA-total FM (all p<0.05). Similar results were noted for WA-total FM%. The total fat mass per unit lean mass (FM/LM) was significantly and inversely associated with the BMD of all studied sites (*P*<0.01), except for the lumbar spine in the men (*P* = 0.065). (Table 2–4).

**Table 2:** Covariate-adjusted mean (SEM) bone mineral density of the lumbar spine by quartiles of each WA-index of FM.

	BMD of the Lumbar Spine						%Diff.	ANCOVA	Z-score	
	Q1		Q2–Q3		Q4			*P*	Linear Regression	
	Mean ± SEM	N	Mean ± SEM	N	Mean ± SEM	N			B	SEM
	g/cm^2^		g/cm^2^		g/cm^2^				mg/cm^2^	mg/cm^2^
Women										
Body weight	0.804±0.010	432	0.860±0.007^†††^	842	0.878±0.011^†††^	421	9.27	<0.001	49.86	6.36***
WA-total FM	0.868±0.009	414	0.851±0.007	845	0.834±0.008^††^	436	−3.93	0.002	−13.02	3.45***
WA-total %FM	0.869±0.009	417	0.850±0.007^†^	842	0.835±0.008^††^	436	−4.01	0.001	−11.24	3.41**
WA-android FM	0.859±0.009	406	0.845±0.007	855	0.855±0.009	434	−0.48	0.144	−0.79	3.44
WA-android %FM	0.855±0.009	411	0.848±0.007	851	0.853±0.009	433	−0.21	0.651	−1.37	3.37
WA-trunk FM	0.862±0.009	409	0.846±0.007	852	0.848±0.009	434	−1.64	0.137	−5.16	3.49
WA-trunk %FM	0.867±0.009	412	0.848±0.007	853	0.841±0.009^†^	430	−2.97	0.018	−7.74	3.45*
WA-limb FM	0.868±0.009	423	0.848±0.007^†^	846	0.839±0.008^††^	426	−3.26	0.005	−12.37	3.20***
WA-limb %FM	0.858±0.009	419	0.853±0.007	848	0.839±0.008	428	−2.24	0.087	−9.27	3.26**
WA-trunk to limb %FM ratio	0.852±0.009	413	0.849±0.007	854	0.853±0.009	428	0.16	0.836	3.43	3.19
WA-FM to LM ratio	0.871±0.009	415	0.849±0.007^†^	844	0.836±0.008^††^	436	−3.98	0.001	−11.53	3.42**
Men										
Body weight	0.924±0.014	168	0.978±0.011^††^	345	1.004±0.015^†††^	169	8.63	<0.001	46.82	10.70***
WA-total FM	0.991±0.013	170	0.972±0.011	339	0.941±0.014^†^	173	−5.02	0.013	−20.28	6.22**
WA-total %FM	0.984±0.013	168	0.974±0.010	342	0.943±0.014	172	−4.18	0.045	−14.80	6.10*
WA-android FM	0.987±0.013	167	0.961±0.011	342	0.976±0.014	173	−1.13	0.146	−7.05	6.09
WA-android %FM	0.966±0.013	167	0.977±0.011	342	0.965±0.014	173	0.07	0.605	−1.15	6.01
WA-trunk FM	0.998±0.013	169	0.966±0.010	340	0.951±0.014^†^	173	−4.70	0.017	−16.25	6.15**
WA-trunk %FM	0.989±0.013	168	0.969±0.010	342	0.954±0.014	172	−3.49	0.127	−11.32	6.10
WA-limb FM	0.991±0.013	170	0.972±0.011	341	0.945±0.013^†^	171	−4.59	0.019	−16.94	5.89**
WA-limb %FM	0.990±0.013	169	0.972±0.010	341	0.947±0.013^†^	172	−4.31	0.034	−14.76	5.92*
WA-trunk to limb %FM ratio	0.967±0.013	170	0.972±0.011	341	0.973±0.013	171	0.67	0.902	4.79	5.59
WA-FM to LM ratio	0.986±0.013	168	0.972±0.011	342	0.947±0.014	172	−3.95	0.065	−16.21	6.09**

FM: fat mass (kg); LM: lean mass (kg); BMD: bone mineral density; WA-: body weight adjusted.

Analysis of covariance (ANCOVA) and linear regression was carried out, controlling for age, education, energy-adjusted (EA-) intake of protein, calcium, and phosphorus, calcium supplements, daily energy expenditure in various physical activities except for sitting and lying, drinking and smoking (for men only), estrogen use, age at menarche, and YSM (for women only), weight (except for weight analysis), and height.

% Diff.: percentage difference = (Q4–Q1)/Q1×100%.

*P*: *P* for group difference.

†, ††, ††† : compared with Q1, *P*<0.05, *P*<0.01, *P*<0.001 (Bonferroni).

* , **, ***: *P* for the linear trend (linear regression), *P*<0.05, *P*<0.01, *P*<0.001.

**Table 3:** Covariate-adjusted mean (SEM) bone mineral density of the total hip by quartiles of each WA-index of FM.

	BMD of the total hip						%Diff.	ANCOVA	Z-score	
	Q1		Q2–Q3		Q4			*P*	Linear Regression	
	Mean ± SEM	N	Mean ± SEM	N	Mean ± SEM	N			B	SEM
	g/cm^2^		g/cm^2^		g/cm^2^				mg/cm^2^	mg/cm^2^
Women										
Body weight	0.771±0.007	432	0.810±0.005^†††^	842	0.830±0.008^†††^	421	7.65	<0.001	46.08	4.66***
WA-total FM	0.820±0.006	414	0.804±0.005^†^	845	0.795±0.006^††^	436	−3.01	0.002	−10.11	2.52***
WA-total %FM	0.819±0.006	417	0.805±0.005	842	0.796±0.006^††^	436	−2.77	0.004	−7.93	2.50**
WA-android FM	0.818±0.006	406	0.803±0.005^†^	855	0.800±0.006^†^	434	−2.31	0.012	−7.71	2.51**
WA-android %FM	0.815±0.006	411	0.804±0.005	851	0.802±0.006	433	−1.61	0.105	−4.89	2.47*
WA-trunk FM	0.820±0.006	409	0.804±0.005^†^	852	0.797±0.006^††^	434	−2.73	0.004	−6.91	2.55**
WA-trunk %FM	0.819±0.006	412	0.803±0.005^†^	853	0.799±0.006^†^	430	−2.49	0.006	−6.02	2.53*
WA-limb FM	0.814±0.006	423	0.806±0.005	846	0.799±0.006	426	−1.86	0.072	−6.87	2.34**
WA-limb %FM	0.814±0.006	419	0.806±0.005	848	0.799±0.006	428	−1.81	0.090	−6.15	2.38*
WA-trunk to limb %FM ratio	0.808±0.006	413	0.805±0.005	854	0.807±0.006	428	−0.11	0.848	1.91	2.34
WA-FM to LM ratio	0.819±0.006	415	0.804±0.005^†^	844	0.797±0.006^††^	436	−2.67	0.005	−8.52	2.50**
Men										
Body weight	0.862±0.010	168	0.922±0.008^†††^	345	0.946±0.011^†††^	169	9.71	<0.001	54.76	7.86***
WA-total FM	0.935±0.010	170	0.914±0.008	339	0.882±0.010^†††^	173	−5.57	<0.001	−21.62	4.53***
WA-total %FM	0.936±0.010	168	0.914±0.008	342	0.885±0.010^†††^	172	−5.38	<0.001	−18.22	4.44***
WA-android FM	0.929±0.010	167	0.906±0.008	342	0.911±0.010	173	−1.98	0.077	−9.40	4.46*
WA-android %FM	0.924±0.010	167	0.911±0.008	342	0.904±0.010	173	−2.16	0.253	−7.48	4.40
WA-trunk FM	0.936±0.010	169	0.908±0.008^†^	340	0.898±0.010^††^	173	−4.07	0.005	−15.51	4.50**
WA-trunk %FM	0.931±0.010	168	0.911±0.008	342	0.896±0.010^†^	172	−3.82	0.018	−13.53	4.46**
WA-limb FM	0.939±0.009	170	0.914±0.008^†^	341	0.881±0.010^†††^	171	−6.20	<0.001	−20.55	4.28***
WA-limb %FM	0.934±0.010	169	0.915±0.008	341	0.888±0.010^††^	172	−4.88	0.001	−19.70	4.30***
WA-trunk to limb %FM ratio	0.901±0.009	170	0.919±0.008	341	0.918±0.009	171	1.83	0.166	8.17	4.09*
WA-FM to LM ratio	0.935±0.010	168	0.913±0.008	342	0.886±0.010^†††^	172	−5.21	0.001	−18.96	4.44***

FM, LM, BMD, WA-: see Table 2.

ANCOVA (analysis of covariance) and linear regression: see Table 2.

% Diff.: percentage difference = (Q4–Q1)/Q1×100%.

*P*: *P* for group difference.

†, ††, ††† : compared with Q1, *P*<0.05, *P*<0.01, *P*<0.001 (Bonferroni).

* , **, ***: *P* for the linear trend (linear regression), *P*<0.05, *P*<0.01, *P*<0.001.

**Table 4:** Covariate-adjusted mean (SEM) bone mineral density of the whole body by quartiles of each WA-index of FM.

	BMD of the Whole Body						%Diff.	ANCOVA	Z-score	
	Q1		Q2–Q3		Q4			*P*	Linear Regression	
	Mean ± SEM	N	Mean ± SEM	N	Mean ± SEM	N			B	SEM
	g/cm^2^		g/cm^2^		g/cm^2^				mg/cm^2^	mg/cm^2^
Women										
Body weight	1.037±0.007	432	1.065±0.005^†††^	842	1.075±0.008^††^	421	3.74	<0.001	29.11	4.60***
WA-total FM	1.082±0.006	414	1.060±0.005^†††^	845	1.043±0.006^†††^	436	−3.65	<0.001	−16.78	2.47***
WA-total %FM	1.085±0.006	417	1.059±0.005^†††^	842	1.041±0.006^†††^	436	−4.05	<0.001	−16.33	2.44***
WA-android FM	1.081±0.006	406	1.058±0.005^†††^	855	1.046±0.006^†††^	434	−3.25	<0.001	−13.48	2.46***
WA-android %FM	1.081±0.006	411	1.056±0.005^†††^	851	1.051±0.006^†††^	433	−2.77	<0.001	−11.87	2.42***
WA-trunk FM	1.089±0.006	409	1.057±0.005^†††^	852	1.043±0.006^†††^	434	−4.21	<0.001	−17.95	2.49***
WA-trunk %FM	1.089±0.006	412	1.056±0.005^†††^	853	1.043±0.006^†††^	430	−4.29	<0.001	−17.61	2.46***
WA-limb FM	1.068±0.006	423	1.061±0.005	846	1.054±0.006	426	−1.26	0.118	−6.59	2.31**
WA-limb %FM	1.069±0.006	419	1.061±0.005	848	1.052±0.006^†^	428	−1.54	0.043	−7.65	2.35**
WA-trunk to limb %FM ratio	1.077±0.006	413	1.056±0.005^††^	854	1.054±0.006^††^	428	−2.16	<0.001	−6.69	2.30**
WA-FM to LM ratio	1.083±0.006	415	1.060±0.005^†††^	844	1.043±0.006^†††^	436	−2.86	<0.001	−16.35	2.45***
Men										
Body weight	1.169±0.010	168	1.188±0.007	345	1.192±0.011	169	1.95	0.148	17.95	7.41*
WA-total FM	1.213±0.009	170	1.185±0.007^†^	339	1.139±0.009^†††^	173	−6.03	<0.001	−29.82	4.19***
WA-total %FM	1.217±0.009	168	1.185±0.007^††^	342	1.140±0.009^†††^	172	−6.29	<0.001	−28.32	4.10***
WA-android FM	1.214±0.009	167	1.177±0.007^†††^	342	1.165±0.009^†††^	173	−4.05	<0.001	−19.95	4.15***
WA-android %FM	1.209±0.009	167	1.181±0.007^†^	342	1.158±0.009^†††^	173	−4.25	<0.001	−18.56	4.10***
WA-trunk FM	1.218±0.009	169	1.180±0.007^†††^	340	1.151±0.009^†††^	173	−5.48	<0.001	−26.90	4.16***
WA-trunk %FM	1.215±0.009	168	1.181±0.007^††^	342	1.150±0.009^†††^	172	−5.31	<0.001	−26.35	4.11***
WA-limb FM	1.208±0.009	170	1.186±0.007^†^	341	1.152±0.009^†††^	171	−5.03	<0.001	−21.89	4.01***
WA-limb %FM	1.208±0.009	169	1.186±0.007	341	1.152±0.009^†††^	172	−4.67	<0.001	−23.49	4.02***
WA-trunk to limb %FM ratio	1.188±0.009	170	1.186±0.008	341	1.177±0.009	171	−0.94	0.555	−3.13	3.87
WA-FM to LM ratio	1.214±0.009	168	1.185±0.007^††^	342	1.141±0.009^†††^	172	−6.00	<0.001	−28.31	4.10***

FM, LM, BMD, WA-: see Table 2.

ANCOVA (analysis of covariance) and linear regression: see Table 2.

% Diff.: percentage difference = (Q4–Q1)/Q1×100%.

*P*: *P* for group difference.

†, ††, ††† : compared with Q1, *P*<0.05, *P*<0.01, *P*<0.001 (Bonferroni).

* , **, ***: *P* for the linear trend (linear regression), *P*<0.05, *P*<0.01, *P*<0.001.

To determine the relationship between fat distribution and BMD, we examined the association of android (abdominal) fat (WA-android FM and WA-android FM %), trunk fat (WA-trunk FM and WA-trunk FM %), and limb fat or %fat with BMD at the three bone sites. In general, limbs and trunk fat or %fat were more closely associated with BMD in the men than in the women. The trunk to limb fat ratio was inversely associated with the BMD of the whole body in the women but not in the men. (Table 2–4).

In addition, we did the sensitivity analyses according to the subgroups stratified by median of age, education level, BMI and dietary energy by gender. There is no significant interaction between these factors and adiposity indices (WA-total FM (or %FM), WA-trunk FM (or %FM) and trunk-to-limb FM ratio) on BMD at the whole body, spine and total hip, except that WA-total FM had significantly greater association with spine BMD in participants with larger BMI (≥median 23.1 kg/m2) than those with lower BMI (<median) (p-interaction = 0.037). (data not shown).

We also compared some baseline characteristics between participants with and without follow-up. There was no significant differences in height, weight, energy intake and expenditure, dietary intake of macronutrients and body composition in both men and women (*P*>0.05). (data not shown).

## Discussion

In this cross-sectional study of Chinese elderly men and women, we found that FM (or %FM) and the FM to LM ratio were inversely associated with BMD after adjustment for mechanical loading effects. Abdominal fat tended to be more significant in the women than in the men, whereas limb fat seemed to be more closely correlated to BMD in the men than in the women. To our knowledge, this study is the first to report that WA-limb fat has a stronger unfavorable association with BMD than abdominal fat or trunk fat in men.

Weight is the predominant predictor of bone mass through its weight-bearing effect [4], [5]. Our data further revealed that body weight was positively related to the BMD of the lumbar spine, total hip, and whole body in both the women and the men. Similar to previous studies [4], [7], [12], [22], when we did not control for body weight we observed significantly positive associations between almost all of the indices of fat and BMD at the lumbar spine and the total hip. However, significantly inverse relations between the total FM (and total %FM) and BMD were found after the mechanical loading effect of body weight was statistically removed. LM has been reported as a predictor of BMD through its mechanical pull on the skeleton [23]. Studies have found that %FM and %LM have a reciprocal relation, but it is unclear whether the risk of a higher %FM for osteoporosis may be due to the effect of a lower %LM [12]. In a study of white and African-American adults, abdominal visceral and subcutaneous fat were negatively related to BMD in the total sample and in various gender-by-race groups after adjusting for lean body mass and other covariates [24]. In our study, the WA-FM to lean mass (LM) ratio had a negative association with BMD, which means that the total FM (or total %FM) was negatively associated with BMD independent of the total LM (or total %LM). Intervention studies [25]–[27] have shown that exercise is associated with a higher bone density and a lower fat mass. Gender, age, menopausal status, and other environmental factors may also confound the association between fat mass and bone mass [28]. Our study analyzed men and women separately, and adjusted for age, YSM (for women only), energy expenditure (excluding sitting and lying), calcium supplements, and other potential confounders. The potential for confounding in our study was thus minimized.

Many studies have reported that fat mass, and especially abdominal fat, is significantly associated with metabolic disorders such as cardiovascular disease (CVD), metabolic syndrome, diabetes, hypertension, and dyslipidemia [29]–[31]. A cross-sectional investigation of 1,631 participants showed an inverse association between bone mass and android fat distribution as measured by anthropometry and DXA after adjustment for BMI, which suggests that android-region or central-region fat deposition is not beneficial and possibly even deleterious to bone density [32]. Several studies have also demonstrated that visceral fat as measured by MRI or CT negatively predicts BMD in both women and men after removing the effect of weight and BMI [24], [33], [34]. Similar to these studies, we found that android and trunk region fat had a similar inverse association with the BMD of the whole body in both genders. In addition, we found that the trunk to limb ratio of %FM had a negative association with the BMD of the whole body in women, but not in men. The negative association between abdominal fat and BMD was consistent in both genders. Unexpectedly, we found that limb fat had an even greater effect on BMD than abdominal fat in the men. A study of 461 Korean adults reported that visceral fat area had a more significant inverse association with BMD in women than in men, but that subcutaneous fat area had no statistically significant relationship with BMD [34]. Another study of 637 white and 544 African-American adults found that subcutaneous adipose tissue was independently and negatively related to BMD, but the correlation was less strong than that with visceral adipose tissue, and the relationships were similar across race and gender groups [24]. A recent study found the accumulation of visceral fat to be the best predictor of metabolic syndrome in women, and the accumulation of subcutaneous fat to be the best predictor in men [35]. Similar to the relationship between fat mass and metabolic related disease, abdominal fat had a more significant and negative effect than limb fat on BMD in the women in this study, but not in the men.

The effect sizes of the relationship between abdominal fat and limb fat with BMD were different in the women and the men. Adipose tissue is known to be more metabolically and biologically active and produces a variety of hormones, inflammatory factors, and cytokines that affect bone metabolism, some of which are site and gender specific. Adiponectin, which is exclusively generated by adipocytes, has been shown to have different effects in men and women, although evidence pertaining to BMD is conflicting. Some studies have found adiponectin to be negatively associated with BMD independent of gender and menopausal status [36]. However, one study reported adiponectin to be inversely associated with total (β = −0.626, *P*<0.001), trabecular (β = −0.696, *P*<0.001), and cortical (β = −1.076, *P* = 0.001) BMD in women, but not in men [37]. Insulin resistance has a negative effect on BMD [38]. Visceral fat, but not total or subcutaneous fat, is the most significant variable correlating with insulin resistance [39], [40]. Although men tend to accumulate more visceral tissue than women, in this study both the absolute and relative android and trunk fat were higher in women than in men. This may partly explain why android and trunk fat had a greater unfavorable effect in women than in men. Leptin may exert dual effects depending on the bone tissue or signaling pathway. The negative influence of leptin on bone formation as effected through the central nervous system pathway may counterbalance these peripheral and positive effects. Leptin predominates when the blood-brain barrier permeability decreases or the serum leptin level rises above a certain threshold [41]. The concentration of leptin is high in subcutaneous fat tissue and low in visceral fat tissue [40], and is two to three fold higher in women than in men, independent of adiposity [41]. Some studies have reported a negative relationship between leptin and BMD in men [42] and a positive effect in postmenopausal women [43]. To sum up, the gender-specific findings of our study could only be partly explained, and further research is needed, especially in terms of the effect of limb fat in men.

We found that the inverse association between FM and BMD was more substantial in the whole body, followed by the total hip and then the lumbar spine in both the women and the men. Other studies have found FM to be inversely associated with cortical density, whereas the association with trabecular density is unclear [44], [45]. The whole body and total hip consist mostly of cortical bone, whereas the lumbar spine mainly consists of trabecular bone. Another reason for our finding may be that the lumbar spine BMD measurements were artificially high due to the presence of osteophytes, disc space narrowing, end-plate sclerosis, and spondylolisthesis and the presence of other structural artifacts such as extraskeletal calcifications [46].

### Limitations

First, we were unable to differentiate between visceral and subcutaneous fat as we used DXA to measure body fat and fat distribution. However, trunk fat and abdominal fat as determined by DXA showed a high correlation with visceral fat as measured by computer tomography (CT) or magnetic resonance imaging (MRI) [47], [48]. A recent study in adolescent girls showed that the trunk %FM and trunk to extremity fat ratio from DXA were significantly more associated with abdominal visceral adipose tissue (r = 0.83 and 0.81 respectively, *P*<0.0001) than abdominal subcutaneous adipose tissue (r = 0.77 and 0.75, *P*<0.0001) [48]. Some researchers have contested that FM should not be adjusted for body weight because of the potential colinearity between the two variables [49]. However, our objective was to explore the influence of body fat and fat distribution on BMD beyond its weight-bearing effect. To avoid colinearity between fat mass and body weight, we included fat mass residuals, rather than fat mass, as our main exposure variable. Next, we did not detect inflammatory cytokines or hormones due to a lack of funds. Third, our study was cross-sectional in nature, and thus we were unable to make inferences of causality between FM and BMD due to the unclear time sequence. Finally, we used the follow-up data of a community-based cohort, which might not be representative of the population as a whole. However, the results and generalisability were unlikely to be greatly affected because participants with different characteristics (such as age, education level, BMI or dietary energy intake) had similar FM-BMD associations. And the higher follow-up rate and similar baseline characteristics between the participants with and without the follow-up suggested low probability of great lost-to follow up bias.

### Conclusion

Total fat is a negative predictor of BMD independent of body weight in the Chinese middle-aged and elderly population. Abdominal fat had a greater unfavorable association with the whole body BMD than limb fat in women, whereas the opposite situation was noted in men. Cortical bone seemed to be more sensitive to body fat than trabecular bone in both genders. Thus, the identified association between fat and fat distribution with BMD is gender and site specific. However, further longitudinal studies are needed to confirm this relation. The results highlight the importance of fat mass as a risk factor for osteoporosis, and provide a rationale for the further exploration of the underlying mechanisms of this effect.
